# Detection of circulating tumor DNA in patients of operative colorectal and gastric cancers

**DOI:** 10.18632/oncotarget.27682

**Published:** 2020-08-25

**Authors:** Takeyuki Suzuki, Tetsutaro Suzuki, Yukino Yoshimura, Mitsunori Yahata, Poh Yin Yew, Tetsuya Nakamura, Yusuke Nakamura, Jae-Hyun Park, Ryota Matsuo

**Affiliations:** ^1^Cancer Precision Medicine, Inc., Kawasaki, Japan; ^2^Yokohama Asahi Chuo General Hospital, Yokohama, Japan; ^3^Itabashi Chuo Medical Center, Tokyo, Japan; ^4^Itabashi Medical System, Tokyo, Japan; ^5^Cancer Precision Medicine Center, Japanese Foundation for Cancer Research, Tokyo, Japan; ^6^Shin-Matsudo Chuo General Hospital, Matsudo, Japan

**Keywords:** ctDNA, early detection, liquid biopsy, mutation, recurrence

## Abstract

Liquid biopsy is a non-invasive tool to examine the genetic profile of tumors by identification of mutated circulating tumor DNA (ctDNA), which is often analyzed by next generation sequencing (NGS) or droplet digital PCR (ddPCR) assay. We first examined the ctDNA mutation in pre-operative plasma samples obtained from 154 colorectal cancer (CRC) and 46 gastric cancer (GC) patients, using the NGS-based panel assay. The overall detection rate of mutated ctDNA was 72.0% (144 of 200 patients), and the panel-based screening identified 207 and 47 mutations from CRC and GC patients, respectively. The ddPCR analysis was then performed on post-operative samples of 77 patients, and detection of mutated ctDNA was earlier than imaging-based diagnosis in all of 6 patients who showed the tumor recurrences after surgery. Our data also revealed that patients with positive post-operation ctDNA level showed significant shorter recurrence-free survival compared to the patients with negative ctDNA level (HR 14.9; 95% CI, 0.7–313.5; *p* < 0.0001). These findings suggested that screening of mutated ctDNA by liquid biopsy aids in identifying the patients at high risk of post-operative recurrence, and serial screening of ctDNA would allow to monitor the response after treatment and/or early detection of tumor recurrence.

## INTRODUCTION

Liquid biopsy is now an emerging approach for the precision oncology, through applications in the detection of cancer cells noninvasively and repeatedly at multiple time points [1]. For example, it can be used to detect the minimal amounts of circulating tumor DNA (ctDNA) isolated from plasma samples of cancer patients. Therefore, genetic alterations in the tumor of individual patient can be rapidly characterized by analyzing the ctDNA, which is released into the blood circulation through apoptosis or necrosis of cancer cells [1]. The cancer-related mutations can be examined even when the tissue biopsy samples are not readily available; likely brain tumors and deeply located solid tumors. In addition, it can minimize the sampling bias caused by multiple tumor lesions or the tumor heterogeneity [2]. Based on these features, liquid biopsy can be utilized for cancer diagnosis, guidance for the matched therapies, monitoring of therapeutic response, and early detection or prediction of tumor recurrence [2–5]. In particular, the detection of mutated ctDNA could serve as a potential biomarker of post-treatment minimal residual disease (MRD) for solid tumors including colorectal, lung, breast, and pancreatic cancer [4, 6–9].

At present, two main methods are widely used for the mutation detection of ctDNA, which are the next generation sequencing (NGS)-based mutation analysis and digital PCR (dPCR) assay [10, 11]. The NGS of ctDNA can broadly identify multiple genetic alterations, while dPCR is restricted to detect the pre-identified mutation and has limited capability of detecting only one mutation per assay [11]. On the other hands, dPCR assay has shown higher sensitivity in detecting variant with relatively lower frequency, compared to the NGS-based mutation analysis [10, 12].

In the present study, we combined these two techniques together for efficient mutation detection and as a monitoring tool for colorectal and gastric cancer patients who received surgery, and addressed the utility of liquid biopsy in monitoring of chemotherapy response and early detection of tumor recurrence after surgery.

## RESULTS

### Study design and blood sample collection

**Figure 1:**
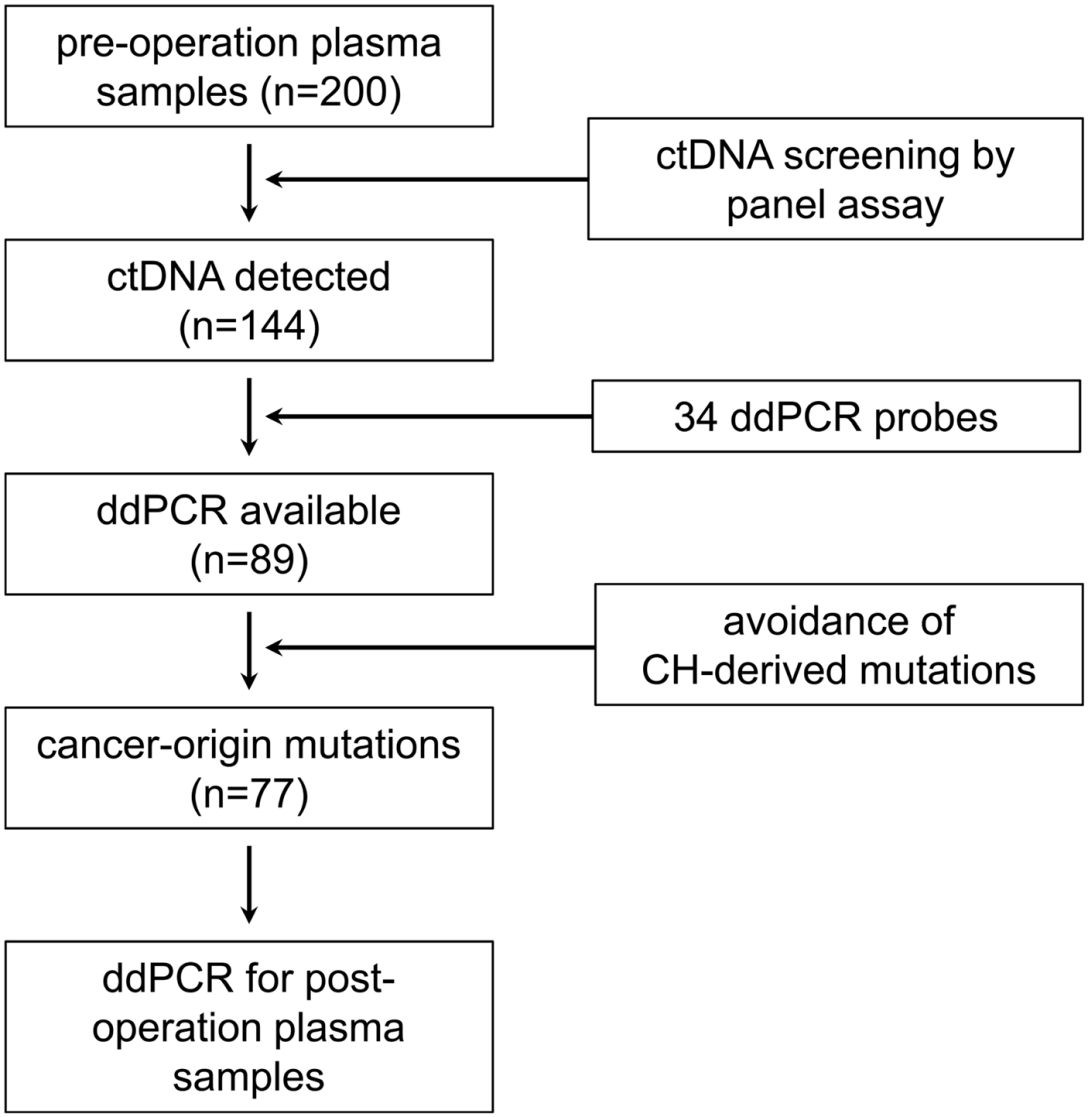
Analysis flow of liquid biopsy in this study. From 200 operative cancer patients (154 CRC and 46 GC), we could detect at least one mutated ctDNA in 144 patients by the panel assay. The 34 validated ddPCR probes were applicable for the detection of mutations in 89 patients. After removal of CH-derived false positive mutations, post-operation plasma samples of 77 patients were finally examined by ddPCR assay.

A total of 200 operative patients of colorectal cancer (CRC, *n* = 154) and gastric cancer (GC, *n* = 46) enrolled this study, and the detailed information of patients is shown in Supplementary Table 1. To examine mutation in ctDNA, we collected peripheral blood at multiple time points including pre-operation, post-operation (average 7.3 days after operation), and every 3 months follow-up – aiming to collect serially until 2 years. Figure 1 describes the experimental flow of this study. Firstly, we examined mutation in ctDNA using a panel assay for 52 cancer-related genes, and each of 144 patients showed at least 1 mutation in the ctDNA. By using 34 droplet digital PCR (ddPCR) probes validated by our own criteria (please see detail in Methods), 89 patients were available for the ddPCR-based detection of ctDNA mutation. Next, we examined genomic DNA (gDNA) from buffy coat of each patient, and removed possible clonal hematopoiesis (CH)-derived mutations which often cause false-positive detections in liquid biopsy [13]. From this filtration criteria, we examined post-operation plasma samples of total 77 patients by the ddPCR assay.

### Detection of mutated ctDNA in pre-operation plasma samples

**Figure 2:**
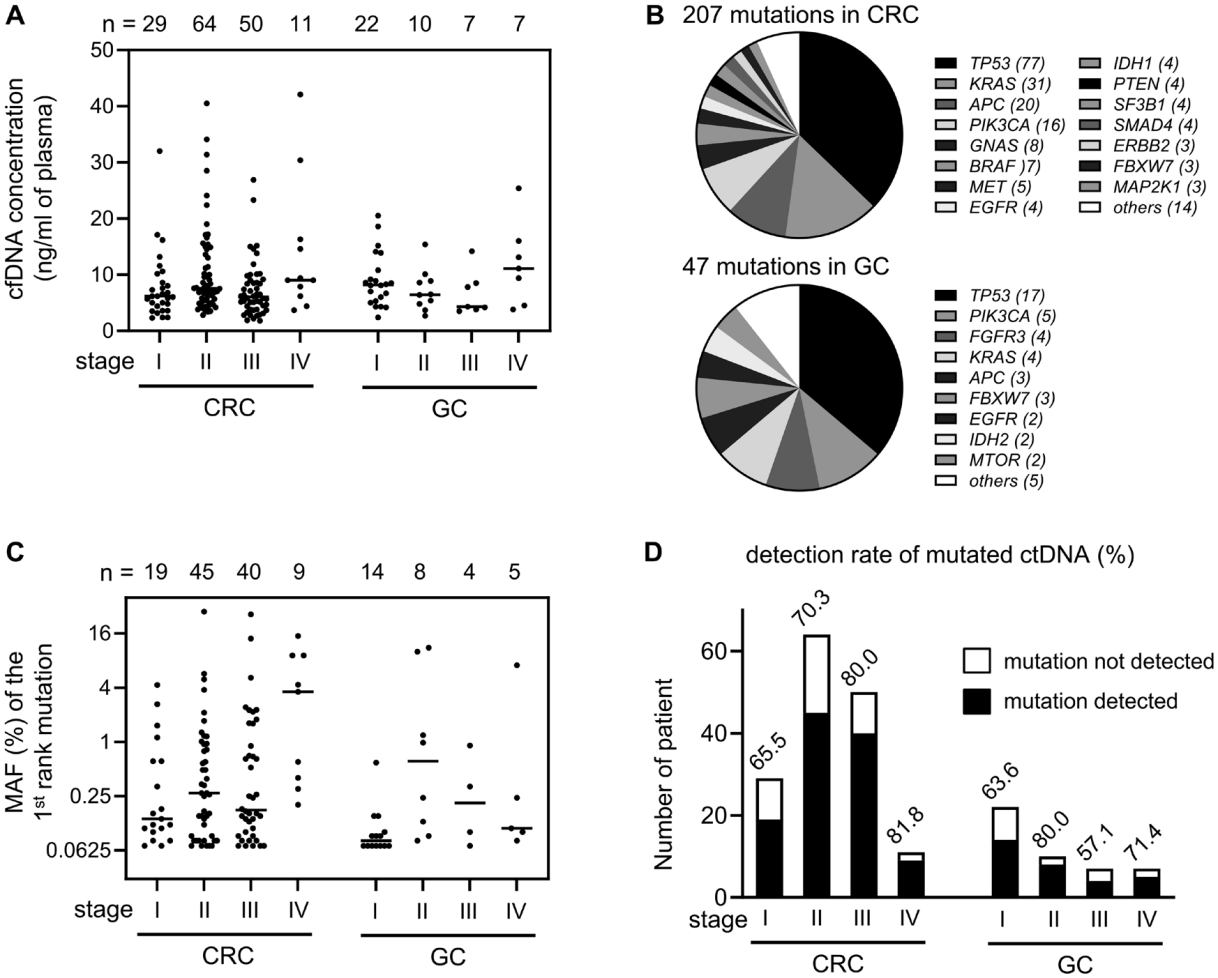
Detection of mutated ctDNA in the pre-operation plasma samples. (**A**) The concentration (ng per 1 ml of plasma volume) of extracted cfDNA was graphed according to cancer type and tumor stage. Each bar in the graph indicates median value. (**B**) Mutation profiles identified in CRC and GC patients. If a patient showed multiple mutations in one gene, only one mutation per gene was counted. (**C**) Each MAF (%) value of the 1st rank mutation was graphed according to cancer type and tumor stage. To show low frequency values clearly, y-axis was transformed by log scale. Each bar in the graph indicates median value. (**D**) Detection rate of liquid biopsy was calculated by counting the patient who showed at least one ctDNA mutation.

From pre-operation plasma samples, we collected 1.8~42.1 ng of cell free DNA (cfDNA) per 1 ml of plasma (Figure 2A). The panel-based mutation screening identified 207 and 47 mutations from 113 CRC and 31 GC patients, respectively. Some patients showed multiple mutations in one gene, probably due to the tumor heterogeneity, so we counted only one mutation per gene per patient for this mutation profiling (Figure 2B). As the results, we found that mutations of *TP53* gene were most frequent in both cancers with the detection rate of 68.1% (77/113) in CRC and 54.8% (17/31) in GC. Other frequent mutations were detected in *KRAS* (27.4%, 31/113) and *APC* (17.7%, 20/113) gene in CRC, and in *PIK3CA* (16.1%, 5/31) in GC.

Next, we examined whether the release of mutated ctDNA is related with the stage of cancer. In the 144 cancer patients who harbored at least one mutated ctDNA, each of mutant allele frequency (MAF) of the 1st rank mutation was graphed according to the cancer stage, which showed a tendency of higher MAF in the later stage of CRC (Figure 2C). The detection rate of mutated ctDNA was gradually increased by tumor stage in CRC, as 65.5%, 70.3%, 80.0%, 81.8% (Figure 2D), which was in concordant with previous report [14]. Such stage-dependent correlation was not clearly found in GC because of limited number of samples per each stage (Figure 2D).

### Monitoring of mutated ctDNA in post-operation plasma samples

By using 34 validated ddPCR probes, we examined the identical mutation in the gDNA isolated from patient’s buffy coat, and consequently found a total of 16 mutations as CH-derived false positive results in 15 patients (Supplementary Table 2). Among them, 3 patients were able to continue with ddPCR analysis by targeting other ctDNA mutations. The ddPCR assays were also validated by cross examination of pre-operative cfDNA samples, which showed a strong correlation in the detection of identical mutations with the NGS-panel assay (Supplementary Figure 1).

**Figure 3:**
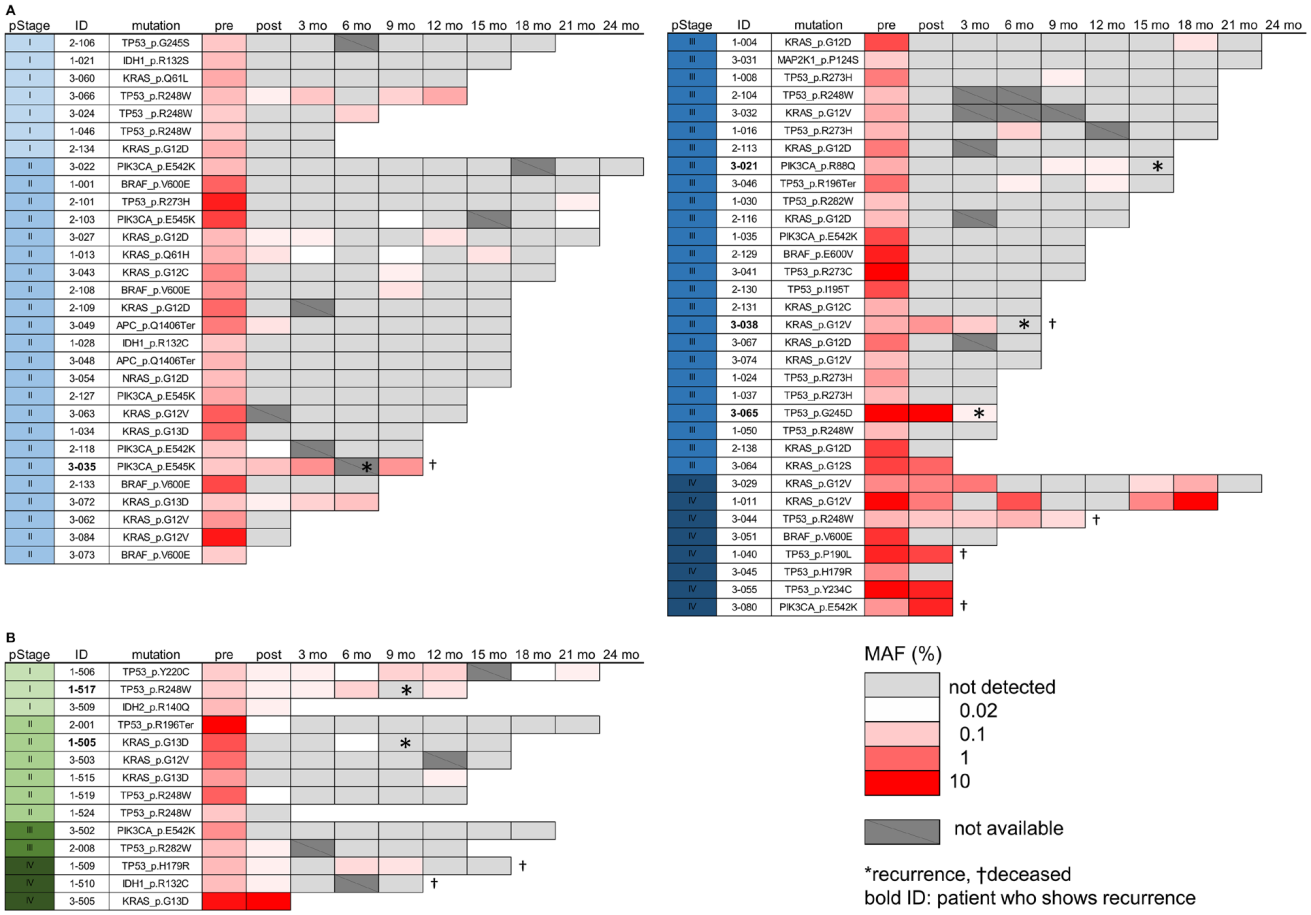
Mutated ctDNA analysis in the post-operation plasma samples. (**A**, **B**) The MAF (%) value of mutated ctDNA was visualized by a heatmap to present data from 63 CRC (A) and 14 GC patients (B). To show low frequency values clearly, the raw MAF values were transformed by log scale, as indicated by a scale bar in the figure. Each table contains information of pStage, detected mutation, and time points of sample collection (pre-operation, post-operation, and 3~24 months after surgery). Not detected; no detection of mutated ctDNA. Not available; plasma sample was not collected or the amount of cfDNA was insufficient for ddPCR assay. Asterisk and cross mark indicate patients of recurrence and deceased, respectively.

In the present study, we could investigate 77 patients (63 CRC and 14 GC) by examination of total 333 plasma samples collected after surgery. The MAF of each mutation of ctDNA in time series was visualized by heat maps (Figure 3). Considering the possibility of very early detection of tumor recurrence, we included even one copy of mutated ctDNA detection as the positive result in ddPCR assays. Until March of 2020, a total of 6 patients have shown tumor recurrences after surgery, as indicated by asterisk marks in Figure 3.

### Early detection of mutated ctDNA before diagnosis of recurrence

**Figure 4:**
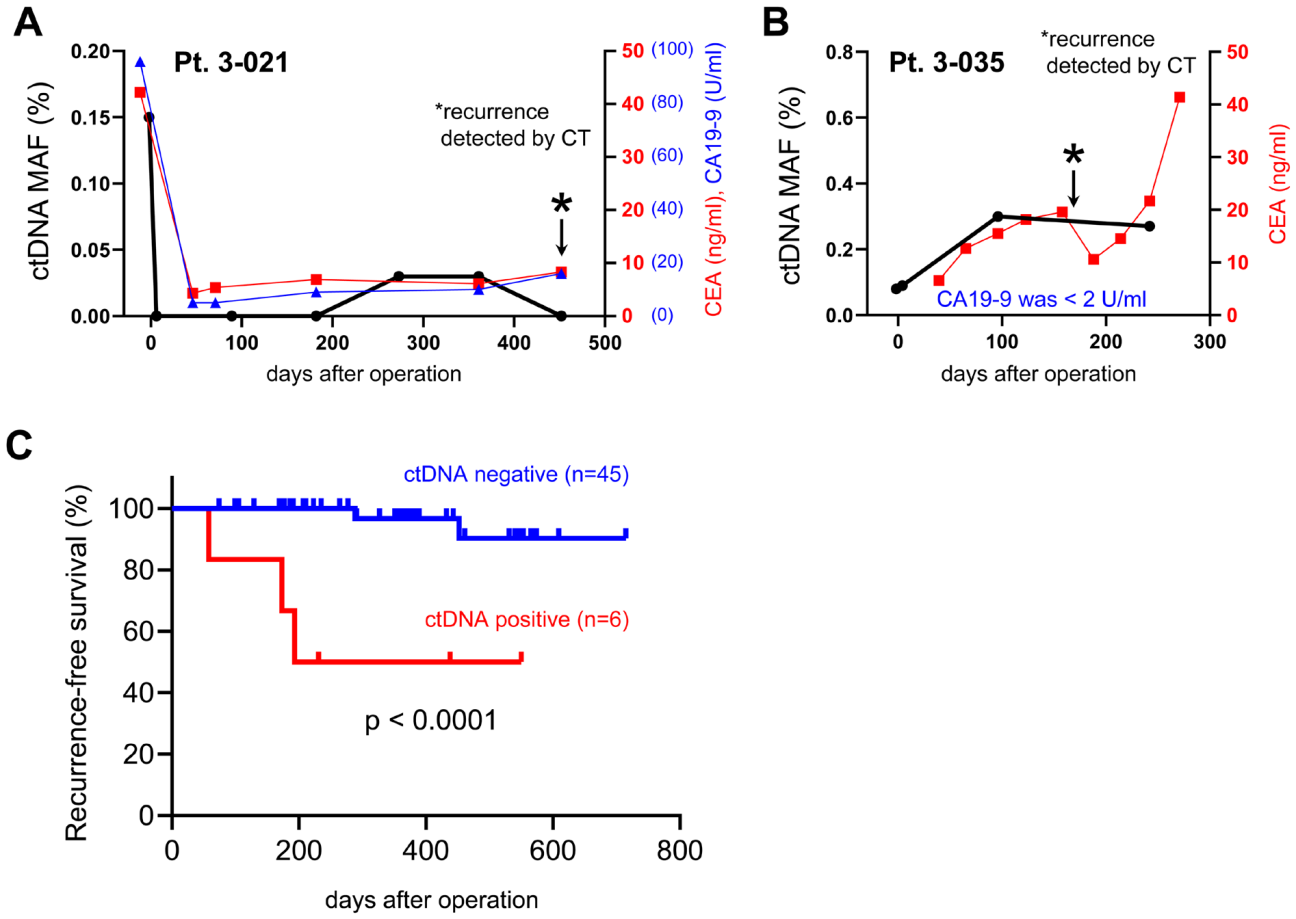
Analysis of mutated ctDNA for early detection of post-operative tumor recurrence. (**A**, **B**) Representative examples from 2 patients (Pt. 3-021 and 3-035) of post-operative recurrence were presented with the values of mutated ctDNA MAF (%), CEA, and CA19-9. (A) Mutated ctDNA (*PIK3CA*, p.R88Q) was re-detected at 273 days after surgery, which was 179 days earlier than diagnosis of recurrence (at 452 days after surgery). (B) Mutated ctDNA (*PIK3CA*, p.E545K) was continuously detected after surgery and increased until 96 days after surgery. The tumor recurrence was diagnosed at 173 days after surgery. Asterisk marks indicated the time point of recurrence. (**C**) Kaplan-Meier curves were generated for RFS analysis of stage II and III of CRC (*n* = 44) or GC (*n* = 7) patients. The ctDNA positive (*n* = 6) patients were selected from whom showed at least 2 copies of mutated ctDNA in the plasma sample of post-operation.

Next, we addressed whether the detection of mutated ctDNA could be used as a predictive biomarker for the early detection of tumor recurrence. In the 6 patients who showed post-operative tumor recurrences, all patients had shown positive detection of mutated ctDNA before the diagnosis of recurrence (Figure 3). For example, a patient (Pt. 3-021) of stage III CRC showed no detection of mutated ctDNA (*PIK3CA*, p.R88Q mutation) after surgery, but the mutation began to be re-detected from 179 days before the diagnosis, while there was no increase of CEA nor CA19-9 levels (Figure 4A). Similar phenomenon was observed in a patient (Pt. 1-505) of stage II GC, mutated ctDNA (*KRAS,* p.G13D) was not detected after surgery while it was re-detected from 81 days before the diagnosis by tumor imaging. Another example is a patient (Pt. 3-035) of stage II CRC, who kept showing mutated ctDNA (*PIK3CA*, p.E545K mutation) until the diagnosis at 173 days after surgery (Figure 4B). Likely this patient, other 3 patients (Pt. 1-517, 3-038, 3-065) also showed continuous detection of mutated ctDNA after surgery (Figure 3), which suggested the possibility of post-operative MRD. Indeed, these patients were not treated with any of adjuvant chemotherapies.

### Detection of MRD and prediction of tumor recurrence by ctDNA analysis

Analysis of post-operative ctDNA has shown promising potential to detect MRD after surgery and to predict high risk of tumor recurrence [6]. By focusing on stage II and III of CRC (*n* = 44) or GC (*n* = 7) patients who were also available for periodic CT diagnosis (median follow up period was 366 days after surgeries), we did recurrence-free survival (RFS) analysis based on the mutated ctDNA level at the early time after surgery; our “post-operation” samples were collected at the end of hospitalization. As shown in Figure 4C, the RFS analysis revealed that the patients who had positive ctDNA level (at least 2 copies of mutated ctDNA) at post-operation showed significant shorter RFS (HR 14.9; 95% CI, 0.7–313.5; *p* < 0.0001) than the patients of negative ctDNA level (Figure 4C). This finding was also replicable if we remove the patients who were treated with adjuvant chemotherapies (Supplementary Figure 2).

### Dynamic change of mutated ctDNA level by chemotherapies

**Figure 5:**
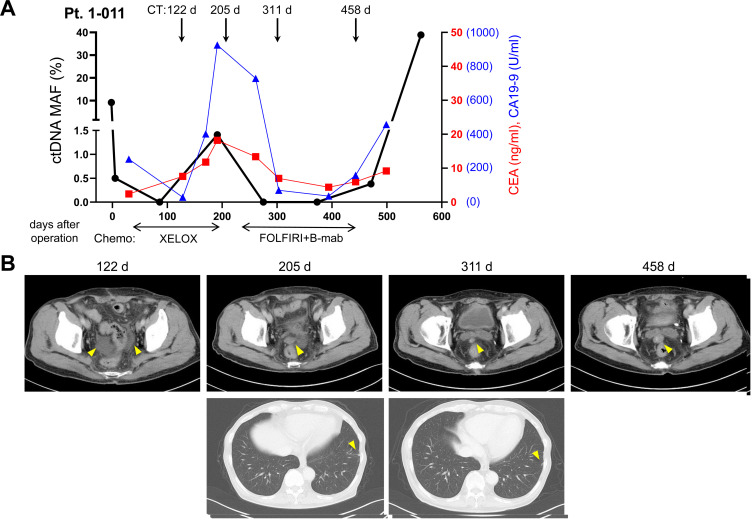
Dynamic change of mutated ctDNA level by chemotherapies. A representative example of stage IV CRC patient (Pt. 1-011) was presented with the values of mutated ctDNA MAF (%), CEA, CA19-9, and CT scan images. (**A**) Level of mutated ctDNA (*KRAS*, p.G12V) was changed according to therapeutic responses to chemotherapy regimen. (**B**) CT scan images during follow up periods were presented. Abdominal CT on 122nd postoperative day showed the first appearance of ascites in the lower pelvic cavity. Abdominal and chest CT on 205th postoperative day showed small nodule of peritoneal dissemination in the pelvic cavity and small lung metastasis in the periphery of left lung. On the 311st postoperative day, pelvic nodule decreased in size but metastatic nodule of the left lung revealed no change. On the 458th postoperative day, pelvic nodule was observed as increased in size.

Another clinical application of liquid biopsy is for the periodical monitoring of therapeutic responses in patient. For example, we observed that a patient (Pt. 1-011) of stage IV CRC showed dynamic change of mutated ctDNA (*KRAS*, p.G12V) by chemotherapies (Figure 5A). After resection of primary colon cancer, the mutated ctDNA level decreased but later it began to increase along with increases of other tumor markers, CEA and CA19-9. Change of chemotherapy regimen from XELOX to FOLFIRI + B-mab caused reduction of pelvic dissemination nodule (Figure 5B), which also resulted in the decrease of mutated ctDNA, CEA, and CA19-9 levels. However, the mutated ctDNA and CA19-9 levels began to increase rapidly after termination of the second chemotherapy, when the increased size of pelvic nodule was observed by CT imaging (Figure 5B). This result implied usefulness of liquid biopsy in the assessment of therapeutic responses, likely other conventional tumor markers.

## DISCUSSION

The incidence of CRC is increasing in many countries, and GC is one of major cancer types in Asian countries. In Japan, CRC and GC are the cancers of 1st and 2nd rank of high incidence [15]. Prognosis of cancer patients mainly depends on the tumor stage at diagnosis, and surgery is one of first line treatment for localized CRC and GC. However, approximately 20~40% of those with stage II or III tumors will develop tumor recurrence in 3 years after curative surgeries, and no consensus biomarker has been identified to predict or detect the post-operative tumor recurrence at early time point [16, 17].

Previous studies have already suggested detection of mutated ctDNA as an accurate prognostic biomarker for the MRD in the resected patients of several tumor types [4, 6–9]. In the presented study, the recurrence rate among patients with mutated ctDNA-positive at the time of post-operation was 50.0% (3 of 6 patients), which was in correlation with a recent ddPCR data in the CRC patients of post-operative recurrences (57.1%, 8 of 14 patients) [7]. Importantly, the study implied that continuous detection of ctDNA in serial samples (called as ‘mutation tracking’) could improve the early prediction of recurrence from 57.1% to 82.4%, suggesting the necessity of periodical follow-up of ctDNA screening to increase the prediction rate for post-operative recurrence [7]. In the surveillance of the resected patients, this approach would be utilized with great potential to prohibit overtreatment in the cured patients by surgery, or on the contrary, undertreatment in the patients who has MRD and higher risk of recurrence.

Another possible application of liquid biopsy is for the detection of recurrence earlier than currently used surveillance tests; such as radiographic imaging, colonoscopy or endoscopy, and blood tumor markers such as CEA and CA19-9. In the previous reports, serial screening of ctDNA enabled to detect tumor recurrences for 81~279 days (median, 167 days) [6] or 3~18 months (median, 11.5 months) [7] earlier than radiological imaging tests in localized CRC resected patients. Although total patient number is small in this study, a patient (Pt. 3-021) of stage III CRC showed 179 days earlier detection of mutated ctDNA than radiological diagnosis of recurrence, even though the patient’s CEA and CA19-9 levels were not increased. Some patients began to show ctDNA-positivity during our late follow-up periods, and there is a possibility to develop tumor recurrence eventually. Careful monitoring would be continued for these patients to enable early diagnosis of recurrences. In addition, it will be important for further investigation of whether ctDNA can be cleared following effective adjuvant chemotherapies, and whether the ctDNA clearance will correlate with patient’s improved overall survival.

The liquid biopsy for ctDNA screening can be conducted using several approaches, including amplicon- or capture-based next-generation sequencing (NGS), digital PCR, and allele-specific PCR. Taking into consideration of the cost-effectiveness in clinical practice, we recruited NGS-panel assay for the first screening of pre-operation sample, then applied ddPCR assays in the analysis of post-operation and serially collected follow-up samples. The ddPCR technique allows absolute quantification and rare allele detection by partitioning individual DNA copies into microdroplets of oil emulsion [18]. Not only for the cost-effectiveness, ddPCR can detect mutated ctDNA with better sensitivity and limit of detection (LOD) level than typical NGS-based assays, which is thereby suitable for very early detection of cancer cells [12]. In the presented study, we could detect ctDNAs of very low MAF as low as 0.02% by using the ddPCR techniques. Some of those patients have not shown any symptom of recurrence, therefore long-time follow-up survey will be necessary to unveil its usefulness as a surrogate risk factor of tumor recurrence.

The *TP53* is most frequently mutated gene in human cancers. Based on the TCGA sequencing data, its mutation rate is 79% in rectum adenocarcinoma, 56% in colon adenocarcinoma, and 48% in stomach adenocarcinoma [19]. Concordantly, we found that mutations of *TP53* gene were most frequently detected in the ctDNA of both CRC and GC patients. Meanwhile, it becomes well known that clonal hematopoiesis of indeterminate potential (CHIP) blood cells also release mutated cfDNAs in the bloodstream, which are unrelated to solid tumors [13]. Ultra-deep sequencings revealed that mutated cfDNAs from the CHIP blood cells were more frequently observed in elder individuals, such as 20% of individuals over 60 years old [20], and up to 53% of cfDNA mutations detected in cancer patients might be derived from the CHIP blood cells [21]. The most frequently CH-derived mutated gene was *DNMT3A*, but mutations of *TP53*, *GNAS*, *SF3B1* genes – which were redundantly detected in this study using gDNA of buffy coat fraction – were also previously reported [20]. Therefore, sequencing of paired gDNA of buffy coat fraction (which includes peripheral blood mononuclear cells) is imperative for the accurate detection of tumor-specific ctDNA, and variants present in both cfDNA and the paired gDNA should be excluded.

A potential limitation of current study is the relatively small sample size of GC patients, especially for the patients with higher stage, compared with CRC patients. In the future, larger sample size could be recruited for GC to obtain more conclusive information and a better understanding for the stage-dependent correlation with ctDNA detection. Another drawback would be the necessity of relatively higher input amount of cfDNA for ddPCR assays, which thus would require collection of more blood volume if the concentration of cfDNA was revealed as very low. However, it is expected that advances in the methodologies for cfDNA stabilization and isolation would enable the test using reduced input of cfDNA.

In conclusion, our findings suggest that screening of ctDNA by liquid biopsy will be useful for a decision-making in early identification of the patients who will be at high risk of post-operative recurrence, and accordingly who should be beneficial from adjuvant chemotherapies. Moreover, serial screening of ctDNA for the resected patient may allow assessment of therapeutic responses to treatment regimen and/or early detection of the post-operative tumor recurrence.

## MATERIALS AND METHODS

### Patients and sample collection

Between March 2018 and March 2020, we collected blood samples from 154 colorectal cancer patients and 46 gastric cancer patients at different time-points; pre-operative, post-operative (at the end of hospitalization, when is average 7.3 days after operation) and every 3 months follow-up – aiming to collect until 2 years. By March 2020, a total of 961 plasma samples were collected from the 200 cancer patients. Those patients are enrolled at Itabashi Chuo Medical Center, Yokohama Asahi Chuo General Hospital, and Shin-Matsudo Chuo General Hospital, which are affiliated in the Itabashi Medical System (IMS) group. This study was approved by the ethics committee in each hospital, and all patients have signed the informed consents.

Peripheral blood samples were collected in 3 × 7 ml EDTA-2Na collection tubes at each time-points. Plasma was separated within 6 hr, which involved a first centrifugation steps of 2,000 ×g at 4°C for 10 min and second step of 16,000 ×g at 4°C for 10 min. At the same time, the buffy coat fraction was transferred to another collection tubes and kept for the detection of CH-derived mutation. Both plasma and buffy coat samples were stored at –80°C until extraction.

### Extraction of cfDNA from plasma and gDNA from buffy coat

Briefly, cfDNAs were isolated from 2.0 ml to 9.6 ml of plasma using MagMAX Cell-Free Total Nucleic Acid Isolation kit (Thermo Fisher Scientific, Waltham, MA) for panel assay or using MagMAX Cell-Free DNA Isolation kit (Thermo Fisher Scientific) for ddPCR assay, according to the manufacturer’s instruction. The cfDNA concentration was quantified using Qubit fluorometer (Thermo Fisher Scientific), while its quality was examined by Bioanalyzer or Tapestation system (Agilent Technologies, Santa Clara, CA).

For the detection of CH-derived mutation, gDNA was isolated from the buffy coat fraction using QIAamp DNA Blood Mini Kit (Qiagen, Germantown, MD). Both quantity and quality of gDNA were examined by NanoDrop (Thermo Fisher Scientific) as well as by Qubit fluorometer (Thermo Fisher Scientific) and Tapestation (Agilent Technologies).

### NGS-based mutation analysis of cfDNA (panel assay)

To identify mutations in the extracted cfDNA samples from cancer patients, we performed NGS-based mutation analysis using Oncomine Pan Cancer Cell Free Assay (Thermo Fisher Scientific) which can examine hotspot mutations of 52 cancer-related genes. The libraries were prepared with input of 8.0 ng–22.6 ng of cfDNA, following manufacturer’s instruction. A maximum of 4 libraries were pooled together and loaded onto the Ion 540 sequencing chip using Ion Chef system (Thermo Fisher Scientific), and then sequenced by Ion GeneStudio S5 Prime System (Thermo Fisher Scientific). The product size, concentration and sequencing output of each experiment were examined. The library which passed internal criteria of QC (quality control) was continued with data analysis.

The obtained sequencing data were then aligned to human reference genome hg19 using Torrent Suite (Thermo Fisher Scientific). Mutation analysis, filtering, and variant annotation were performed with Ion Reporter ver 5.6 (Thermo Fisher Scientific) using an automatic workflow of Oncomine TagSeq Pan-Cancer Liquid Biopsy w2.0 (Thermo Fisher Scientific).

### Monitoring of ctDNA by ddPCR

To monitor the response after surgery, representative ctDNA mutations of 77 patients were examined by ddPCR. The examined ctDNA mutations were basically selected according to following criteria; i) mutation detected with the highest frequency in patient, ii) common mutation detected in several patients, iii) registered mutation of ≥ 1% frequency in the gene, according to the COSMIC database (https://cancer.sanger.ac.uk/cosmic). Briefly, 34 ddPCR probes matching with the detected ctDNA mutations were purchased from Bio-Rad or Thermo Fisher Scientific (Supplementary Table 3). To detect the possibility of CH-derived mutations, the pre-operative gDNA for these patients were examined while the cfDNA was used as positive control to validate the detection reactivity of ddPCR probe. Only the validated ddPCR probes were used to examine the post-operative cfDNA samples at each time-point.

For the ddPCR reaction, a total of 20 ul PCR mixtures were prepared by adding 2x ddPCR supermix (Bio-Rad, California, US) for probes without dUTP, 20x primer/probe reagent (Bio-Rad or Thermo Fisher Scientific), and 8.4–22.0 ng of cfDNA in distilled water. Positive control (gDNA) and negative controls (distilled water) were included for each test, and the PCR reaction mixture was then portioned into droplets using QX-200 Droplet Generator (Bio-Rad). The generated droplets were transferred to a PCR plate, and the reaction was performed using C1000 Touch Thermal cycler (Bio-Rad), according to the manufacturer’s protocol. Each assay was optimized for annealing temperatures combined with the manufacturer’s recommended primers and probes concentrations (Supplementary Table 3). Finally, data analysis was performed using the QuantaSoft Software version 1.7.4 (Bio-Rad).

### Analysis of clinical data

Serum CEA and CA19-9 level were measured at pre-operative, post-operative and every 3 months until 2 years as shown in graphs. In addition, chest and abdominal CT scan preferably using contrast agent were also performed in the same time course of CEA and CA19-9 level measurement. The pathological stage (pStage) was judged by UICC-TNM criteria based on the pathological results of the resected specimen.

For stage II and III CRC or GC patients, the RFS (recurrence-free survival) analysis was performed by dividing 2 groups, according to detection of mutated ctDNA (at least 2 copies) in the sample of “post-operation” which was collected at the end of hospitalization (average 7.3 days after operation).

### Statistical analysis

GraphPad Prism ver 8 (GraphPad Software, San Diego, CA) was used for Kaplan-Meier curves and a log-rank (Mantel-Cox) test of the RFS analysis. The *p* value < 0.05 was considered as statistically significant.

## SUPPLEMENTARY MATERIALS





## Abbreviations

cfDNA: cell free DNA
CH: clonal hematopoiesis
ctDNA: circulating tumor DNA
CRC: colorectal cancer
ddPCR: droplet digital PCR
GC: gastric cancer
MAF: mutant allele frequency
MRD: minimal residual disease
NGS: next generation sequencing
RFS: recurrence-free survival
