# Supplementary material for: Detection of circulating tumor DNA in patients of operative colorectal and gastric cancers
Source: Oncotarget. 2020 Aug 25;11(34):3198–207. doi: 10.18632/oncotarget.27682 (PMC7456613; doi:10.18632/oncotarget.27682)
Supplement: Supplementary file 1 [file oncotarget-11-3198-s001.pdf]

Detection of circulating tumor DNA in patients of operative colorectal and gastric cancers

SUPPLEMENTARY MATERIALS

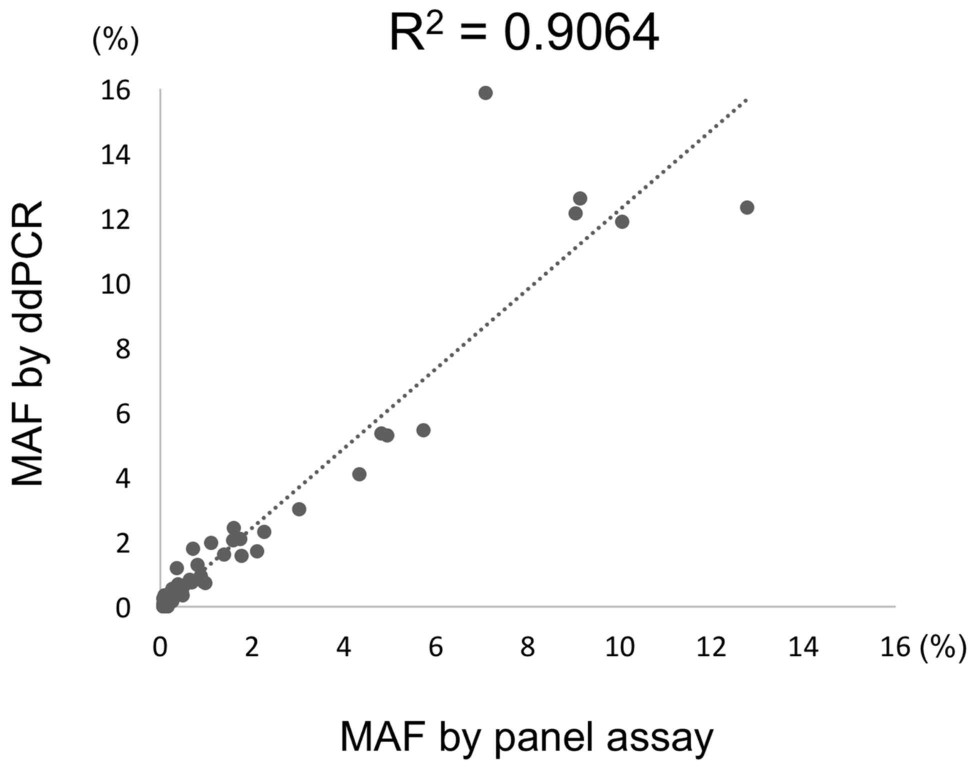

Supplementary Figure 1: Correlation of mutation detection trait from panel assay and ddPCR. For the validation of ddPCR probes, the MAF (%) values of identical mutation from the NGS-based panel assay (x-axis) and ddPCR (y-axis) were compared using plasma samples of pre-operation.

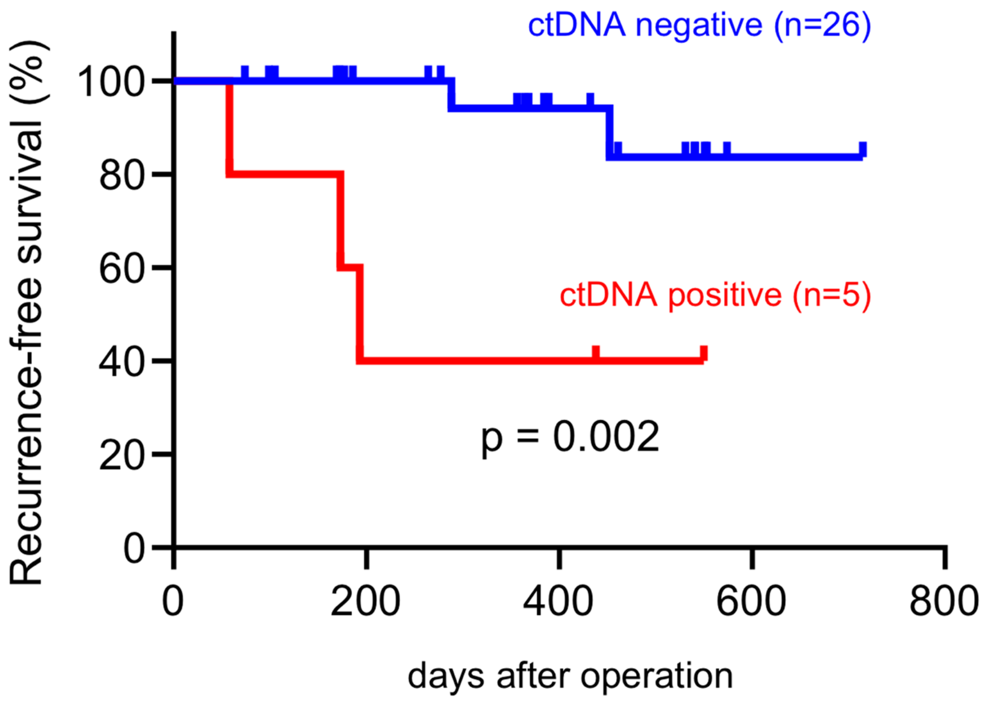

Supplementary Figure 2: RFS analysis for the patients who were not treated with adjuvant chemotherapies. Focusing on the patients who were not treated with adjuvant chemotherapies, Kaplan-Meier curves were re-generated for RFS analysis of stage II and III of CRC (n = 28) or GC (n = 3) patients. The ctDNA positive (n = 5) patients, who showed at least 2 copies of mutated ctDNA in the plasma sample of post-operation, showed significant shorter RFS (HR 9.8; 95% CI, 0.7–130.1; p = 0.002) than the patients of negative ctDNA level.

Supplementary Table 1: Summary of detected ctDNA mutations. See Supplementary Table 1

Supplementary Table 2: List of CH-derived mutations

pt. ID	mutation	MAF (%) in plasma (panel)	MAF (%) in buffy coat (dPCR)
1-012	SF3B1_p.K700E	0.240	0.102
1-013	GNAS_p.R201H	3.770	2.163
1-018	TP53_p.R280G	4.280	2.426
1-045	GNAS_p.R201H	0.070	0.027
1-051	SF3B1_p.K700E	2.620	0.800
1-516	TP53_p.I195T	0.150	0.140
1-520	TP53_p.G245D	0.150	0.230
2-132	GNAS_p.R201H	0.340	0.284
3-030	TP53 _p.R248W	0.150	1.371
3-036	TP53_p.R273L	1.010	0.600
3-036	TP53_p.Y234C	0.330	0.166
3-054	SF3B1 _p.K700E	0.576	0.332
3-058	IDH2_p.R140Q	0.900	0.671
3-066	GNAS_p.R201H	0.610	0.238
3-069	TP53_p.P190L	1.520	1.563
3-510	FGFR3_p.R248C	0.090	0.059

Supplementary Table 3: List of ddPCR probes

Gene	Annotation	Nuelcotide change	Annealing temperature (°C)	Manufacturer	Assay ID
APC	p.Q1406Ter	c.4216C>T	57	Bio-Rad Laboratories, Inc.	dHsaMDS2513750
BRAF	p.V600E	c.1799T>A	60	Thermo Fisher Scientific Inc.	Hs000000004_rm
FGFR3	p.R248C	c.742C>T	60	Bio-Rad Laboratories, Inc.	dHsaMDV2516906
GNAS	p.R201H	c.602G>A	55	Bio-Rad Laboratories, Inc.	dHsaMDV2516796
IDH1	p.R132C	c.394C>T	55	Bio-Rad Laboratories, Inc.	dHsaMDV2010053
IDH1	p.R132S	c.394C>A	55	Bio-Rad Laboratories, Inc.	dHsaMDV2516816
IDH2	p.R140Q	c.419G>A	55	Bio-Rad Laboratories, Inc.	dHsaMDV2010057
KRAS	p.G12D	c.35G>A	55	Bio-Rad Laboratories, Inc.	dHsaMDV2510596
KRAS	p.G12S	c.34G>A	55	Bio-Rad Laboratories, Inc.	dHsaMDV2510588
KRAS	p.G12V	c.35G>T	60	Thermo Fisher Scientific Inc.	Hs000000050_rm
KRAS	p.G13D	c.38G>A	55	Bio-Rad Laboratories, Inc.	dHsaMDV2510598
KRAS	p.Q61H	c.183A>C	55	Bio-Rad Laboratories, Inc.	dHsaMDV2010133
KRAS	p.Q61L	c.182A>T	55	Bio-Rad Laboratories, Inc.	dHsaMDV2010101
KRAS	p.G12C	c.34G>T	55	Bio-Rad Laboratories, Inc.	dHsaMDV2510584
MAP2K1	p.P124S	c.370C>T	55	Bio-Rad Laboratories, Inc.	dHsaMDS400568388
NRAS	p.G12D	c.35G>A	55	Bio-Rad Laboratories, Inc.	dHsaMDV2010095
PIK3CA	p.E542K	c.1624G>A	55	Bio-Rad Laboratories, Inc.	dHsaMDV2010073
PIK3CA	p.E545K	c.1633G>A	57	Thermo Fisher Scientific Inc.	Hs000000086_rm
PIK3CA	p.R88Q	c.263G>A	55	Bio-Rad Laboratories, Inc.	dHsaMDV2510558
SF3B1	p.K700E	c.2098A>G	55	Bio-Rad Laboratories, Inc.	dHsaMDS576883070
TP53	p.G245D	c.734G>A	55	Bio-Rad Laboratories, Inc.	dHsaMDV2510542
TP53	p.G245S	c.733G>A	57	Bio-Rad Laboratories, Inc.	dHsaMDV2516746
TP53	p.H179R	c.536A>G	55	Bio-Rad Laboratories, Inc.	dHsaMDV2010125
TP53	p.I195T	c.584T>C	57	Bio-Rad Laboratories, Inc.	dHsaMDV2516898
TP53	p.P190L	c.569C>T	55	Bio-Rad Laboratories, Inc.	dHsaMDS339194743
TP53	p.R248W	c.742C>T	60	Thermo Fisher Scientific Inc.	Hs000000094_rm
TP53	p.R273C	c.817C>T	55	Bio-Rad Laboratories, Inc.	dHsaMDV2510538
TP53	p.R273H	c.818G>A	60	Thermo Fisher Scientific Inc.	Hs000000095_rm
TP53	p.R273L	c.818G>T	55	Bio-Rad Laboratories, Inc.	dHsaMDV2510504
TP53	p.R280G	c.838A>G	58	Bio-Rad Laboratories, Inc.	dHsaMDS2515638
TP53	p.R282W	c.844C>T	55	Bio-Rad Laboratories, Inc.	dHsaMDV2516902
TP53	p.Y220C	c.659A>G	57	Bio-Rad Laboratories, Inc.	dHsaMDV2510536
TP53	p.R196Ter	c.586C>T	55	Bio-Rad Laboratories, Inc.	dHsaMDV2010121
TP53	p.Y234C	c.701A>G	55	Bio-Rad Laboratories, Inc.	dHsaMDV2516900
